# Effects of Seed Size and Frequency on Seed Dispersal and Predation by Small Mammals

**DOI:** 10.3390/biology13050353

**Published:** 2024-05-16

**Authors:** Jiming Cheng, Min Zhang, Xingfu Yan, Chao Zhang, Jinfeng Zhang, Yonghong Luo

**Affiliations:** 1 College of Biological Science and Engineering, North Minzu University, Yinchuan 750021, China; chengjiming@mails.ccnu.edu.cn; 2 School of Life Sciences, Central China Normal University, Wuhan 430079, China; minzhang@mails.ccnu.edu.cn (M.Z.); zhangchao2023@mails.ccnu.edu.cn (C.Z.); 3 Key Laboratory of Ecological Protection of Agro-Pastoral Ecotones in the Yellow River Basin, National Ethnic Affairs Commission of the People’s Republic of China, Yinchuan 750021, China; 4 College of Life Sciences, Qinghai Normal University, Xining 810016, China; 20231034@qhnu.edu.cn; 5 School of Ecology and Environment, Inner Mongolia University, Hohhot 010000, China

**Keywords:** frequency dependence, population regeneration, prey on, *Quercus wutaishanica*, scatter-hoarding, seed mass, seed size

## Abstract

**Simple Summary:**

Animal-mediated seed dispersal plays a crucial role in the regeneration of plant populations and the stability of forest ecosystems. In this study, we used the tagging method to measure the effects of seeds of different sizes at different frequencies on seed fate and dispersal distance. We found that rodents consumed more small seeds in situ, dispersed and scatter-hoarded more large seeds, and dispersed large seeds over longer distances. Rodents showed a negative frequency dependence for small seeds and a positive frequency dependence for large seeds on being eaten in situ. Moreover, rodents showed a negative frequency dependence for large seeds and a positive frequency dependence for small seeds on being eaten after removal and scatter-hoarding. These findings provide insights into the frequency dependence of animal predation on seed size, animal-mediated seed dispersal, and the regeneration of plant populations.

**Abstract:**

Frequency-dependent predation is common in predator–prey interactions. Size is an important characteristic of seeds and is crucial in the regeneration stage of plant seeds. However, the frequency dependence of animal predation on seed size has not been reported. In this study, we conducted a field experiment and used different sizes of Liaodong oak (*Quercus wutaishanica*) seeds to test the frequency dependence of intraspecific seed size selection in rodents. We used the number ratio of large to small seeds as the frequency. The results show that the rate of small seeds being eaten in situ was significantly higher than that of large seeds (*p* < 0.05). The rates of different-sized seeds being eaten after removal decreased with increasing frequencies, and there was no significant difference between frequencies except for 1:9 and 9:1. The rates of large seeds being scatter-hoarded were significantly higher than those of small seeds at different frequencies (*p* < 0.05). The eating distances after removal of large seeds were significantly longer than those of small seeds at the same frequencies (*p* < 0.05). Furthermore, the scatter-hoarding distances of large seeds were significantly longer than those of small seeds at three frequencies (1:9, 3:7, and 9:1) (*p* < 0.05). That is, rodents consumed more small seeds in situ, dispersed and scatter-hoarded more large seeds, and dispersed large seeds over longer distances. Rodents exhibited a negative frequency dependence for small seeds and a positive frequency dependence for large seeds on being eaten in situ. Moreover, rodents exhibited a negative frequency dependence for large seeds and a positive frequency dependence for small seeds on being eaten after removal and scatter-hoarding. These results reveal the frequency dependence of rodent selection on seed size and provide new insights into animal-mediated seed dispersal and the regeneration of plant populations.

## 1. Introduction

Zoochory is an important seed dispersal strategy. Rodents mediate the dispersal of various seed species, which plays important roles in seedling recruitment, community demographics, and species diversity [1,2]. Plants and scatter-hoarding rodents have formed a dual relationship over the long period of their co-evolution [3]. Studies have shown that scatter-hoarding rodents are both predators and dispersers of seeds; they scatter-hoard a portion of the harvested seeds to cope with food shortages. If scatter-hoarded seeds are not retrieved by rodents, the seeds are highly likely to survive [4,5]. Although some of the seeds (produced by plants) are eaten, the goals of spreading the species and providing a better place for germination are achieved. The return gained by the plants is far higher than their previous investment in terms of evolutionary significance.

The effect of seed size on rodent-mediated seed dispersal has attracted much attention in the literature [3,6,7]. Seed size plays an important role in plant life history, especially seed dispersal, germination, seedling establishment, and survival [8,9,10]. Large seeds are better adapted to adverse biotic and abiotic factors, including competition, drought, destruction intensity, and nutrient limitations [11,12]. Therefore, it is widely accepted that large seeds are more beneficial to seed regeneration. In addition, the size (or the nutritional value) of seeds plays a “keystone” role in the hoarding behavior of predators and dispersers [13,14]. Plants regulate the dispersal, seed-feeding, and hoarding behaviors of rodents by manipulating seed size [15,16,17]. Generally, scatter-hoarding rodents prefer to hoard large or high-nutritional-value seeds and consume relatively small seeds [3,8,18,19]. The dispersal distance of a seed is correlated with seed size; large seeds are generally dispersed over longer distances [19,20,21]. However, some reports indicate that large seeds may have a lower survival rate due to higher predation pressure before dispersal and higher detection and pilferage rates after hoarding (because of stronger odors) [22,23,24]. How seed size affects seed dispersal thus remains controversial.

Frequency-dependent selection has significant effects on the dynamics between predators and prey. In prey populations, positive frequency-dependent selection may lead to monomorphic phenomena or convergent evolution, and negative frequency-dependent selection can maintain phenotypic diversity [25,26,27]. Frequency dependence can explain short-term evolutionary direction changes and is also important in long-term evolution [28]. Prey frequency may influence the intensity and direction of frequency-dependent selection. Predators may consume more common prey than rarer prey at low prey densities, while they may consume rarer prey at high prey densities [29,30]. Similar to the relationship between predators and prey, the relationship between rodents and plant seeds can be used as a model to test the effects of the frequency-dependent selection of specific seed phenotypes on plant population dynamics. Previous studies have focused on how seed predators and dispersers select seeds based on specific seed characteristics (e.g., seed thickness and nutritional value) [9,31,32,33]; however, few studies have explored the effects of different seed frequencies and specific seed characteristics (e.g., seed size) on the consumption and hoarding behaviors of rodents, and how this selection affects the regeneration, distribution, and population dynamics of plants.

Liaodong oak (*Quercus wutaishanica*) is one of the main dominant species (referring to numerical superiority) in warm temperate deciduous forests in China, and it is a species of ecological and economic value [10]. The seeds are generally mature and shed from late August to the end of September [34]. Under natural conditions, an extremely low proportion (<0.1%) of seeds become germinated and established seedlings [3,35]. The seeds suffer from heavy predation by rodents, which may cause a significant bottleneck in their population regeneration. However, whether seed sizes and frequencies and these interactions affect seed predation and dispersal by rodents is still unclear. This experiment aimed to explore the effects of different seed sizes and frequencies on the predation and dispersal of *Q. wutaishanica* seeds by rodents, and promote our understanding of the role that animal-mediated seed dispersal/predation plays in the regeneration of plant populations.

## 2. Materials and Methods

### 2.1. Study Sites

The study area was located in the Liupanshan National Nature Reserve (35°15′–35°41′ N, 106°09′–106°30′ E). The district is located in a temperate subhumid area with a continental monsoon climate [10]. The annual precipitation is 676 mm, 60% of which is concentrated from July to September, and the annual evaporation volume is 1426 mm [10]. The annual average temperature is 5.8 °C, and the average temperatures in the hottest month (July) and the coldest month (January) are 17.4 °C and 7.0 °C, respectively [10]. The soil is mainly gray-brown, and the zonal vegetation is temperate coniferous forest, with *Pinus armandii* as the dominant species, and secondary deciduous broad-leaved forest, with *Q. wutaishanica*, *Populus davidiana*, *Betula platyphylla*, and *B. albo-sinensis* as the dominant species. Additionally, there are many shrub communities, including *Q. wutaishanica*, *Hippophae rhamnoides*, *Prunus davidiana*, *Ostryopsis davidiana*, and *Cotoneaster acutifolius*. The artificial forests in this area mainly include *Larix principis-rupprechii* and *Pinus tabuliformis* forests.

The study site was located in the oak forest area of the Longtan Forest Farm, with a slope of 35° [34]. Most of the Liaodong oak plants in this community are more than 2.5 m high, and the total coverage of the community is above 90%. The shrubs and small trees, besides Liaodong oak, include *Prunus armeniaca*, *Crataegus kansuensis*, *C. multiflorus*, *Lonicera ferdinandii*, *Spiraea pubescens*, and *Fargesia nitida* [34]. The herbs mainly include *Smilax china*, *Thymus vulgaris*, *Paeonia lactiflora*, *Rubia membranaca*, *Fragaria orientalis*, and *Agropyron cristatum*. There are *Sus scrofa*, *Phasianus colchicus*, *Chrysolophus pictus*, *Pucrasia macrolopha*, *Perdix dauurica*, *Coturnix coturnix*, and other herbivorous animals; they occasionally consume *Q. wutaishanica* seeds. The rodents mainly include *Mus musculus*, *Niviventer confucianus*, *Apodemus agrarius*, *Microtus fortis*, and *Sciurotamias davidianus*; and the rodents that mainly consume and disperse seeds are *N. confucianus* and *S. davidianus*.

### 2.2. Seed Collection and Marking

Liaodong oak seeds were collected from 30-year-old Liaodong oak trees in the Longtan Forest Farm on 18 September 2017, and returned to the laboratory. We selected healthy (i.e., not worm eaten or moldy) Liaodong oak seeds to store in a 4 °C refrigerator. The differences in length, diameter, and fresh weight between the different-sized seeds were significant (*p* < 0.05) (Table 1).

A small hole of 0.5 mm in diameter was drilled at the base of each seed with an electric drill. Then, a copper wire (7 cm in length) wrapped in a plastic protective layer was put through each hole. A red plastic label (2.5 × 1.2 cm), which recorded the information on the seed size, frequency, and location coordinates, was attached to the copper wire (total mass: 0.2 ± 0.002 g; *n* = 100) [36]. The labels on the seeds eaten by animals were discarded at the consumption point, while the labels on seeds that were hoarded after dispersal remained attached. Therefore, the seed fate types could be distinguished by searching for the labels during the field survey [9].

### 2.3. Seed Experiment

On 1 November 2017, five shrub transects of Liaodong oak were selected on the west side of the ditch, each with a transect length and width of approximately 90 m and 50 m, respectively. The distance between the two adjacent transects was kept above 30 m. Three seed release points were selected from the bottom to the top of the mountain in each transect, and the distance between two adjacent release points was more than 20 m. The seeds were divided into two types: large and small. The large and small seeds were mixed at five frequencies—9:1 (108:12), 7:3 (84:36), 5:5 (60:60), 3:7 (36:84), and 1:9 (12:108)—each with 3 replicates. The seeds of the two types were evenly mixed into a circle with a 50 cm radius (with the label pointing outside and the seeds pointing toward the circle) and marked near the drop point to facilitate later inspection [6]. The number of seeds used in the experiment was 120 (seed) × 3 (repetition) × 5 (frequency) = 1800 seeds.

Each seed release point was checked on the 1st, 2nd, 3rd, 4th, 5th, 6th, 14th, 21st, and 30th days after seed release, and the seed labels dropped within a 30 m radius of the release points were recorded [17]. The seed fates were recorded based on [17,31] as follows: (1) eaten in situ: rodents consumed the seeds at the release point, and the labels were abandoned and fell at the release point; (2) eaten after removal: rodents consumed the seeds during the dispersal process, and the labels were left on the path at a certain distance from the release point; (3) scatter-hoarded: the seeds were dispersed and hoarded under soil, litter, and trees; (4) missing: the seeds were not found after being removed from the seed release point, and the fate of the seeds could not be determined. We also recorded the seed dispersal distance, including the eaten distance after removal (EDAR) and the scatter-hoarding distance (SHD). The hoarded and consumed seeds were found by seeking labels within a radius of 25 m around the center of each plot, and the seed label information, the distances between the dispersed seeds and the center of the corresponding release point, and the number of seeds at the consumption and hoarding points were recorded [3,17].

### 2.4. Data Analysis

We calculated the percentage of seeds of each fate type (eaten in situ, eaten after removal, scatter-hoarded, and missing) versus the initially released seeds. Then, we used generalized linear models (GLMs) to analyze the effects of seed size, frequency, and their interaction on seed fate and dispersal distance. We used seed size, frequency, and their interaction as explanatory variables and seed fate and dispersal distance (eaten and scatter-hoarded) as dependent variables, plotted as random effects. We fitted generalized linear models for seed fate with count variables (family = Poisson; link = log) and those for dispersal distance with Gaussian variables (family = Gaussian; link = identity) in the “lmer4” package [37]. We used least significant difference (LSD) analysis to examine the percentage of seeds with different fates and the dispersal distances of seeds in SPSS (Version 21.0). A square root transformation was applied if the data did not satisfy a normal distribution. We performed the statistical analysis with R 4.3.2 (R Development Core Team; http://r-project.org; accessed on 1 February 2024) and SPSS (Version 21.0) [10,37]. The normality test and the data standardization were conducted using SPSS 21.0. All the data are expressed as means ± standard deviations (Std.). We used SigmaPlot (Version 12.5) to produce all the figures.

## 3. Results

### 3.1. Effects of Different Sizes and Frequencies on Seed Fates

Seed size and frequency had significant effects on being eaten in situ, eaten after removal, and scatter-hoarded (*p* < 0.05). The interaction between seed size and frequency had no significant effect on being eaten in situ, eaten after removal, or scatter-hoarded (*p* > 0.05). Seed size, frequency, and their interaction had no significant effect on the missing seed fate category (*p* > 0.05) (Table 2).

**Figure 1:**
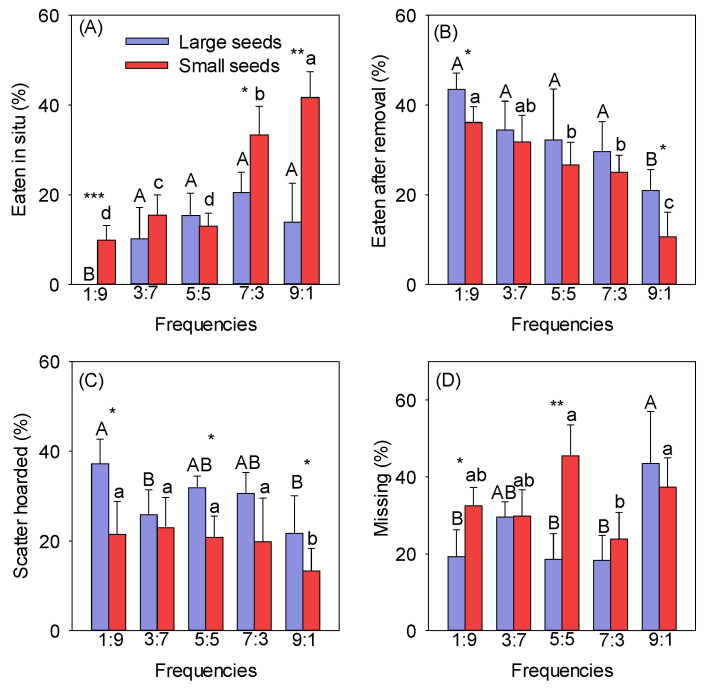
Effect of frequency on the eaten in situ (**A**), eaten after removal (**B**), scatter-hoarded (**C**), and missing (**D**) fates of different-sized seeds. Different uppercase letters indicate significant differences between large seeds at different frequencies, and different lowercase letters indicate significant differences between small seeds at different frequencies. * *p* < 0.05, ** *p* < 0.01, and *** *p* < 0.001 indicate significant differences between seeds of different sizes at the same frequency.

The rates at which different-sized seeds were eaten in situ gradually increased with increasing frequencies (Figure 1A). The rate at which large and small seeds were eaten in situ was lowest at the 1:9 frequency (the mean values for the large and small seeds were 0 and 9.88%, respectively). The rate at which small seeds were eaten in situ was highest at the 9:1 frequency (the mean value was 45.00%) and was significantly greater than at the other frequencies (*p* < 0.05). The rate at which large seeds were eaten in situ was highest at the 7:3 frequency (the mean value was 22.69%), but there were no differences between the 3:7, 5:5, 7:3, and 9:1 frequencies (Figure 1A). The rate at which small seeds were eaten in situ was significantly higher than that for large seeds at the 1:9, 7:3, and 9:1 frequencies (*p* < 0.05) (Figure 1A). The rate at which different-sized seeds were eaten after removal decreased with increasing frequencies, and there was no significant difference between frequencies, except for 1:9 and 9:1 (Figure 1B). The rate at which seeds were eaten after removal was highest at the 1:9 frequency (the mean values for the large and small seeds were 43.50% and 36.11%, respectively) and lowest at the 9:1 frequency (the mean values for the large and small seeds were 20.95% and 10.67%, respectively) (Figure 1B). The rates at which seeds of different sizes were scatter-hoarded decreased with increasing frequencies (Figure 1C). The rate at which large seeds were scatter-hoarded was highest at the 1:9 frequency (the mean value was 37.22%), and that of small seeds was highest at the 3:7 frequency (the mean value was 22.93%). The rate at which seeds were scatter-hoarded was lowest at the 9:1 frequency (the mean values for the large and small seeds were 21.70% and 13.33%, respectively) (Figure 1C). The rates at which large seeds were scatter-hoarded at different frequencies were significantly higher than those for small seeds at the 1:9, 5:5, and 9:1 frequencies (*p* < 0.05) (Figure 1C).

### 3.2. Effects of Different Sizes and Frequencies on Seed Dispersal Distance

**Figure 2:**
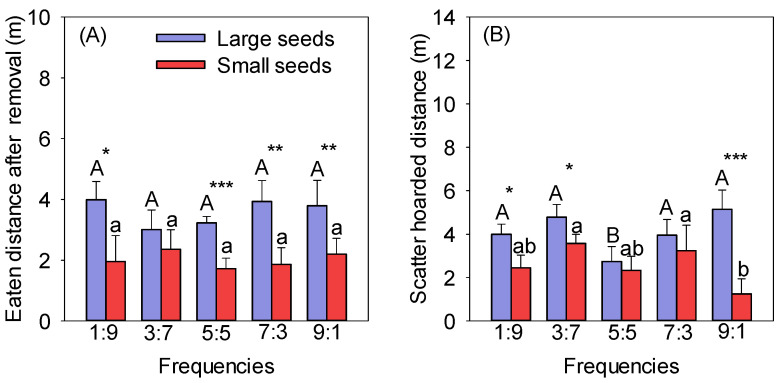
Effect of frequency on the EDARs (**A**) and SHDs (**B**) of different-sized seeds. Different uppercase letters indicate significant differences between large seeds at different frequencies, and different lowercase letters indicate significant differences between small seeds at different frequencies. * *p* < 0.05, ** *p* < 0.01, and *** *p* < 0.001 indicate significant differences between seeds of different sizes at the same frequency.

The EDARs of large seeds were significantly longer than those of small seeds at the same frequencies, except for the 3:7 frequency (*p* < 0.05). There was no significant difference in the EDAR of the same seeds at different frequencies (Figure 2A). The SHDs between seeds of different sizes were significantly different, except for at the 5:5 and 7:3 frequencies, and those of large seeds were significantly higher than those of small seeds at the other three frequencies (1:9, 3:7, and 9:1) (*p* < 0.05) (Figure 2B). The SHD of the large seeds at the 5:5 frequency was significantly lower than at the other frequencies, and there was no significant difference between the other frequencies (*p* < 0.05) (Figure 2B). The SHDs of large seeds were higher than those of small seeds at different frequencies and significantly higher at the 1:9, 3:7, and 9:1 frequencies (Figure 2B). The minimum SHD for small seeds was 1.25 m at the frequency of 9:1, significantly lower than at the 3:7 and 7:3 frequencies (*p* < 0.05) (Figure 2B).

The eaten and hoarded distances of large seeds had the highest distributions in the 3–5 m and 5–10 m distance groups and the lowest distributions in the <1 m and >10 m distance groups (Table A1). The eaten and hoarded distances of small seeds were mainly distributed in the <1 m and 1–3 m distance groups (Table A1).

### 3.3. Size of Consumption and Hoarding Points

The largest predation points of large seeds contained two seeds, comprising nine consumption points; the largest predation points of small seeds contained four seeds, comprising only two consumption points (Table A2). The seed consumption points of rodents mainly contained one seed, whether large or small, accounting for more than 95% of all consumption points. The consumption points of other sizes were relatively few, accounting for no more than 5%. The largest mixed predation point contained 20 seeds. Two, nine, and ten consumption points contained four, three, and two seeds, respectively (Table A2). The largest mixed hoarding point had 72 seeds. Three and two mixed hoarding points contained two and three seeds, respectively (Table A2).

**Figure 3:**
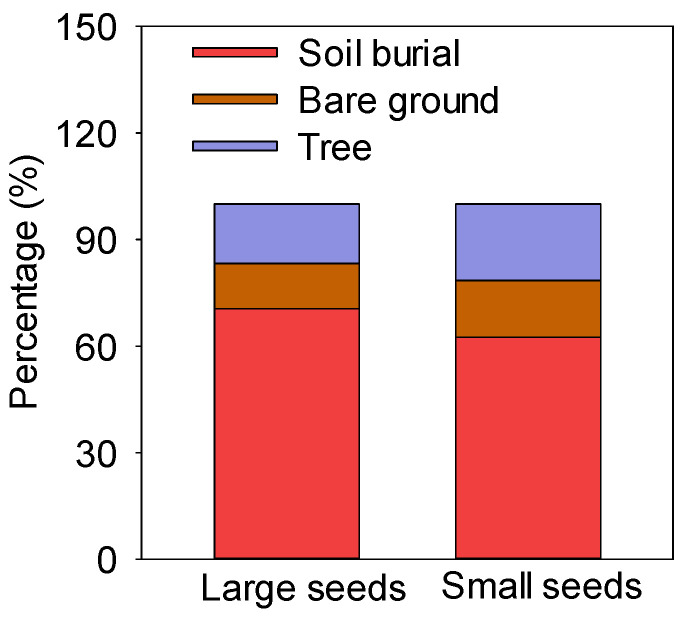
Hoarding methods for large and small seeds.

There were three hoarding methods for seeds of different sizes, i.e., soil burial, exposure on the bare ground, and hoarding in trees (including at the base of trees and abandoned nests) (Figure 3). The rates of soil burial, exposure on the bare ground, and hoarding in trees of large seeds were 70.52%, 12.77%, and 16.71%, respectively, while those in trees of small seeds were 62.55%, 16.00%, and 21.45%, respectively. The soil burial rate of large and small seeds was more than 60% (Figure 3).

## 4. Discussion

The dispersal and predation of plant seeds by rodents could closely link the seed and seedling stages of adult plant life-history processes, and is an important ecological process that affects plant population and community dynamics [13,17]. Scatter-hoarding animals have significant effects on the predation, dispersal, and hoarding of plant seeds, thus affecting plant population regeneration, community composition, and ecosystem diversity [38,39]. Plants generally produce seeds of different sizes, and the seeds do not mature synchronously, which can result in different sizes and frequencies of seeds for selection by animals. There is an important relationship between the nutritional value of foods and the frequency at which animals choose those foods [17,32]. Seed size reflects seed quality and is often used as a standard for evaluating animal predation selection [10,40,41]. Different seed sizes and frequencies are crucial for rodent-mediated seed dispersal.

The rate at which large seeds were eaten in situ was significantly lower than that for small seeds at different frequencies, while the rates at which large seeds were eaten after removal and scatter-hoarding were higher than those for small seeds. In addition, the eaten distance after removal (EDAR) and scatter-hoarding distance (SHD) for large seeds were higher than those for small seeds. These results are consistent with the conclusions of many researchers; i.e., rodents tend to disperse and cache large seeds with a high nutritional value, disperse large seeds over longer distances, and consume small seeds with a relatively low nutritional value in situ [3,10,19,31]. There may be a trade-off in rodents between the nutritional benefits of dispersing and hoarding different-sized seeds and the time spent, energy expended, and predation risk. Rodents disperse and cache large seeds to obtain the same nutritional value in the food returns when they search for fewer hoarding points to retrieve the hoarded seeds [10]. Hoarding small seeds with fewer nutrients may be insufficient to compensate for the time spent, energy expended, and predation risk for rodents during dispersal and hoarding [8].

Researchers believe that predators may stabilize prey populations via positive frequency dependence [26]. Prey frequency may alter the intensity and direction of frequency-dependent selection. Predators tend to consume more common prey at lower prey frequencies and rarer prey, which are different from common prey, at higher prey frequencies [42]. This benefits the survival of rare prey species [26,43]. In contrast, negative frequencies destabilize predator populations and may lead to the eventual extinction of rare prey species [26]. Our results show that rodents exhibited a negative frequency dependence for small seeds and a positive frequency dependence for large seeds on being eaten in situ. Moreover, rodents exhibited a negative frequency dependence for large seeds and a positive frequency dependence for small seeds on being eaten after removal and scatter-hoarded. Rodents need a higher energy input to consume and cache large seeds after dispersal, and the energy input increases as the number of dispersed seeds increases [10,17]. Therefore, rodents consume a portion of the seeds as an energy supplement, preferentially consuming small seeds [3]. Once all the small seeds have been consumed, they begin to consume the large seeds. The energy consumption for dispersing small seeds is lower, and the energy already stored by a rodent and a small amount of food supplement could enable the dispersal of seeds [17]. Therefore, rare large seeds may have better fitness before dispersal, while small seeds have better fitness after dispersal. As such, large and small seeds eventually have the same fitness, resulting in the coexistence of large and small seeds. This agrees with the results reported by Cao et al. [7].

Hoarding animals often face the risk of pilferage by intra- or interspecific competitors when hoarding food. Scatter-hoarding food in a large space can reduce the density of cache points, thus reducing the chance of competitors discovering cached food (i.e., optimal hoarding spacing) [9,10,44]. Both large and small Liaodong oak seeds mainly undergo single-grain predation and hoarding, which conforms to the prediction of the “optimal hoarding model” [17,44]. Low seed density reduces intraspecific competition, and effectively avoids the seeds being found by the predators; and the seeds have a higher probability of survival, which is more beneficial to the regeneration of the plant population [10,27,34]. Although rodents face a higher predation risk and energy input when scatter-hoarding a large number of seeds to retrieve the cached seeds, larder-hoarding points can be easily pilfered by other animals. Therefore, scatter-hoarding can reduce the risk of all the hoarded food being pilfered due to larder-hoarding to ensure a basic food supply during a food shortage (i.e., the pilfering avoidance hypothesis) [45]. Although there were some larder-hoarding points in our study (including hoarding points containing 72, 65, and 20 seeds), these seeds were cached in an abandoned nest nearly 2 m above the ground to exclude competitors who could not climb trees. Rodents may need to scatter-hoard seeds multiple times. As such, rodents may have dispersed seeds for the first time during our investigation, leading to the larder-hoarding phenomenon [17,46].

Seeds have a high risk of being pilfered when rodents hoard them in shrubs, and seeds discarded on the bare ground may be eaten or dispersed again by other animals [17]. Therefore, soil burial is the safest hoarding method and may be a long-term hoarding strategy. Our results show that large and small seeds are mainly buried in soil, which reflects the results reported by some researchers [9,20]. Soil burial can reduce the risk of pilferage and pathogen infection and also avoid the increase in costs of memorizing, managing, and re-excavating new hoarding points caused by re-hoarding.

This study found that both seed size and frequency have significant effects on seed fates, but the interaction between seed size and frequency has no effects on seed fates and dispersal distance. According to our results, when the frequency difference of different-sized seeds is large (1:9 and 9:1), it has a significant impact on the fate of seeds, but the impact is not significant at other frequencies. This may be the small number of repetitions in our experiments. In the future, we could add the number of repetitions in the same year, conduct long-term seed tracking between different years, and add multiple plant species, etc.

## 5. Conclusions

In this study, we used seeds of different sizes and frequencies from the same species to explore rodent consumption and hoarding behavior preferences with seed size asymmetry. Our results indicate that rodents consume more small seeds in situ, disperse and scatter-hoard more large seeds, and disperse large seeds over longer distances. Rodents exhibited a negative frequency dependence for small seeds and a positive frequency dependence for large seeds on being eaten in situ. Moreover, rodents exhibited a negative frequency dependence for large seeds and a positive frequency dependence for small seeds on being eaten after removal and scatter-hoarding. Seed size and plant seed production were negatively correlated, which partly explains the coexistence of seeds of different sizes. It is still necessary to periodically track the fates of large and small seeds over a long period and thus determine the seed fitness and the outcomes of future natural selection.

## Figures and Tables

**Table 1:** Characteristics of *Q. wutaishanica* seeds of different sizes.

Seed Traits	Mean ± Std.		Mann–Whitney Test
	Large Seeds	Small Seeds	
Length (mm)	21.78 ± 1.20	16.65 ± 1.33	*p* < 0.05
Diameter (mm)	15.95 ± 0.85	12.13 ± 0.70	*p* < 0.05
Fresh mass (g)	3.05 ± 0.38	1.46 ± 0.27	*p* < 0.05

Note: Std. indicates standard deviation (*n* = 100).

**Table 2:** Difference analysis of effects of different seed sizes and frequencies on seed fates.

Variable	Eaten In Situ			Eaten after Removal			Scatter-Hoarded			Missing		
	df	F	*p*	df	F	*p*	df	F	*p*	df	F	*p*
SS	1	16.268	**<0.001**	1	7.406	**0.010**	1	7.771	**0.008**	1	2.554	0.126
F	4	11.229	**<0.001**	4	4.823	**0.003**	4	4.257	**0.006**	4	4.671	0.098
SS × F	4	2.522	0.056	4	1.101	0.369	4	0.895	0.476	4	0.567	0.743

Note: SS: seed size; F: frequency. Bold characters indicate a significant difference (*p* < 0.05).

## Data Availability

Dataset available upon request from the authors.

## Appendix A

**Table A1:** Effect of frequency on the dispersal distances of seeds of different sizes.

Seed Fate	Distance Group	Large Seeds					Small Seeds				
		9:1 (0.9)	7:3 (0.7)	5:5 (0.5)	3:7 (0.3)	1:9 (0.1)	9:1 (0.9)	7:3 (0.7)	5:5 (0.5)	3:7 (0.3)	1:9 (0.1)
Eaten	<1 m	10 (11.49%)	15 (18.52%)	14 (20.90%)	6 (13.22%)	5 (33.33%)	4 (44.44%)	15 (48.39%)	20 (41.67%)	21 (35.59%)	47 (40.51%)
	1–3 m	19 (21.84%)	24 (29.63%)	10 (14.93%)	12 (32.43%)	3 (20.00%)	4 (44.44%)	12 (38.71%)	22 (45.83%)	20 (33.90%)	65 (56.03%)
	3–5 m	12 (13.79%)	16 (19.75%)	28 (41.97%)	13 (35.14%)	2 (13.33%)	0 (0.00)	2 (12.90%)	3 (6.25%)	18 (30.51%)	2 (1.72%)
	5–10 m	36 (41.38%)	20 (24.69%)	14 (20.90%)	5 (13.51%)	4 (26.67%)	1 (11.11%)	2 (12.90%)	3 (6.25%)	0 (0.00)	1 (0.86%)
	>10 m	10 (11.49%)	6 (7.41%)	1 (1.50%)	1 (2.70%)	1 (6.67%)	0 (0.00)	0 (0.00)	0 (0.00)	0 (0.00)	1 (0.86%)
Hoarded	<1 m	1 (0.95%)	28 (28.57%)	14 (22.95%)	8 (16.67%)	5 (27.78%)	2 (50.00%)	6 (30.00%)	8 (20.00%)	1 (1.41%)	55 (52.88%)
	1–3 m	21 (20.00%)	2 (2.06%)	24 (39.34%)	10 (20.83%)	3 (16.67%)	2 (50.00%)	4 (20.00%)	20 (50.00%)	35 (49.30%)	22 (21.15%)
	3–5 m	39 (37.14%)	55 (57.14%)	9 (14.75%)	18 (37.50%)	0 (0.00)	0 (0.00)	9 (45.00%)	10 (25.00%)	30 (42.25%)	13 (12.50%)
	5–10 m	38 (36.19%)	20 (20.61%)	12 (19.67%)	11 (22.92%)	5 (27.78%)	0 (0.00)	0 (0.00)	3 (7.50%)	3 (4.23%)	12 (11.54%)
	>10 m	6 (5.71%)	2 (2.06%)	2 (3.28%)	1 (2.08%)	5 (27.78%)	0 (0.00)	1 (5.00%)	0 (0.00)	2 (2.82%)	2 (1.92%)

## Appendix B

**Table A2:** The sizes of the predation and hoarding points of large and small seeds, alone and mixed.

Predation/Hoarding Size			1	2	3	4	8	13	20	65	72
Eaten after removal	Large seeds	Number	235	9	-	-	-	-	-	-	-
		Percentage (%)	96.31	3.69	-	-	-	-	-	-	-
	Small seeds	Number	203	4	1	2	-	-	-	-	-
		Percentage (%)	96.67	1.90	0.48	0.95	-	-	-	-	-
Scatter-hoarded	Large seeds	Number	243	3	-	-	-	-	-	-	-
		Percentage (%)	98.78	1.22	-	-	-	-	-	-	-
	Small seeds	Number	140	-	-	-	-	-	-	-	-
		Percentage (%)	100.00	-	-	-	-	-	-	-	-
Mixed large and small seeds	Eaten after removal	Number	-	10	9	2	-	-	1	-	-
		Percentage (%)	-	45.45	40.91	9.09	-	-	4.55	-	-
	Hoarding after removal	Number	-	3	2	-	1	1	-	1	1
		Percentage (%)	-	33.33	22.22	-	11.11	11.11	-	11.11	11.11
